# Implementing a Graduate Certificate Program in Cardiovascular Epidemiology: The Jackson Heart Study

**DOI:** 10.3390/ijerph13010026

**Published:** 2015-12-22

**Authors:** Brenda W. Campbell Jenkins, Clifton Addison, Gregory Wilson, Lavon Young, Regina Fields, Clevette Woodberry, Marinelle Payton

**Affiliations:** Jackson Heart Study, School of Public Health, Jackson State University, 350 West Woodrow Wilson Drive, Suite 2900B, Jackson, MS 39213, USA; brenda.w.campbell@jsums.edu (B.W.C.J.); gregory.wilson@jsums.edu (G.W.); lavon.young@jsums.edu (L.Y.); regina.fields@jsums.edu (R.F.); clevette.woodberry@jsums.edu (C.W.); marinelle.payton@jsums.edu (M.P.)

**Keywords:** cardiovascular epidemiology, graduate training, Jackson heart study

## Abstract

The Jackson Heart Study (JHS) is committed to providing opportunities for expanding the understanding of the epidemiology, diagnosis, prevention, and treatment of cardiovascular diseases. The JHS Graduate Training and Education Center (GTEC) has initiated the Daniel Hale Williams Scholar (DHWS) program where students are afforded the opportunity to interact with epidemiologists and other biomedical scientists to learn to identify, predict, and prevent cardiovascular disease using the Jackson Heart Study data. This study describes the structured programs developed by JHS GTEC seeking to alleviate the shortage of trained professionals in cardiovascular epidemiology by training graduate students while they complete their academic degrees. The DHWS program provides: (1) an enrichment curriculum; (2) a learning community; (3) quarterly seminars; and (4) a Summer Institute. Students attend enrichment activities comprising: (1) Applied Biostatistics; (2) Cardiovascular Disease Epidemiology; (3) Social Epidemiology; (4) Emerging Topics; and (5) Research Writing. Training focuses on developing proficiency in cardiovascular health knowledge. The DHWS program is a unique strategy for incorporating rigorous academic and career-focused training to graduate students and has enabled the acquisition of competencies needed to impact cardiovascular disease management programs.

## 1. Introduction

In the State of Mississippi, the prevalence and incidence rates for obesity and cardiovascular diseases are the highest in the nation [1,2]. According to the CDC, cardiovascular disease (CVD), a class of diseases that affect the heart and blood vessel system, is the leading cause of mortality among all Americans. In 2002, the Jackson Heart Study (JHS) reported that cardiovascular deaths in African Americans were 22% higher in Mississippi than the rest of the United States [3]. The prevalence of obesity, a risk factor for CVD, is particularly high in Mississippi. According to the Robert Wood Johnson Foundation, Mississippi had an obesity rate of 35.4 percent in 2015 [4]. Among African American adults in the JHS, 32% were characterized as mostly overweight and 53% were classified as obese. Only 12.1% of females and 18.0% of males in the JHS had normal weight with a BMI of 25 kg/m^2^ and below [5]. These statistics are alarming not only to African Americans in the United States, but especially to Mississippians. The estimated annual medical cost of obesity in the U.S. was $147 billion in 2008; the annual medical costs for people who are obese were $1429 higher than those of normal weight [6]. Treatment of these diseases accounts for about $1 of every $6 spent on health care in this country [7]. Reduction in the prevalence and incidence of these diseases has become a major part of innovative health improvement and health education programs [8] to advance the health status of Mississippians, particularly African Americans.

The JHS began in 2000 as a partnership among three institutions, Jackson State University, University of Mississippi Medical Center, and Tougaloo College. Jackson State University developed and managed the Coordinating Center, University of Mississippi Medical Center managed the Examination Center, and Tougaloo College had the responsibility of operating the Undergraduate Training Center. Three exams have been completed so far; Exam 1 was conducted during 2000–2004, and 5301 participants were recruited; Exam 2 was conducted from 2005–2008; and Exam 3 extended from 2009–2012. Data collection continues through annual follow-up activities. A substantial number of publications is available presenting research conducted by JHS investigators including staff at the three partner institutions, as well as external investigators who operate through various Working Groups and Vanguard Centers. In 2013, the structure of the JHS was changed from three centers to five centers with Jackson State University contracted to operate a new Graduate Training and Education Center (GTEC) and a new Community Outreach Center (CORC), University of Mississippi Medical Center contracted to operate the Coordinating Center and the Field Center, and Tougaloo College contracted to manage the Undergraduate Training and Education Center. The primary responsibility of GTEC is to train graduate students in cardiovascular epidemiology through the DHWS Program. Additional initiatives are needed to address the paucity of African-American public health and biomedical scientists in Mississippi, as well as to increase the number of students who pursue graduate education in the health sciences in Mississippi. In order to improve disease detection, treatment, and prevention in the African-American community that comprises about 80% of the population in Mississippi and are disproportionately affected by CVD, there is a need for an innovative approach to encourage and train African-American scholars to aspire to become health professionals so that they can become involved in doing research, treating patients, and developing prevention strategies for the population at risk where health disparities persist [9]. To effectively address the problems related to cardiovascular disease, there is a great need for skilled providers who can effectively deliver health-related services that meet the social, cultural, and linguistic needs of the African American community [10]. Training culturally-competent public health and biomedical professionals, the focus of the JHS GTEC, can help improve health outcomes and quality of care in the long run, and can contribute to the elimination of racial and ethnic health disparities.

In August 2013, Jackson State University was awarded a contract from the National Heart, Lung, and Blood Institute (NHLBI) of the National Institutes of Health (NIH) to implement a JHS GTEC. GTEC is designed to implement a robust education training program by applying evidence-based approaches to train graduate students in the social, behavioral, and medical sciences. The JHS has an objective to build capacity among its communities, and one focus is on developing core competencies from which students in the biomedical and public health disciplines can develop the knowledge, skills, abilities, and other professional attributes that are required to perform successful service in cardiovascular disease management.

The mission of the JHS GTEC at Jackson State University is to provide opportunities in public health and biomedical research techniques for scholars to improve their capacity in community-based research and practice. To achieve the goals, GTEC initiated the DHWS program to prepare graduate students at the masters’ and doctoral levels utilizing mentoring and training in public health with a focus on the underserved and at- risk populations in Mississippi. The program promotes public health and biomedical science education and provides enrichment opportunities to equip GTEC scholars with the capacity to contribute to the prevention of CVD disease and disability. As a service goal, GTEC’s operations promote the development of technical expertise, advocacy skills, and translational capabilities in the scholars to support the mission of its internal stakeholders (NHLBI, Jackson State University, and the JHS) and external stakeholders (local, national, and global communities, public agencies, and the private sector) [11].

For many minority students who specialize in public health and biomedical sciences, the current university graduate curriculum does not prepare them to immediately assume a position/career in their chosen field upon graduation. Some educators have expressed alarm that after the long process on the way to graduating with a professional degree, students still may not have acquired skills that are comprehensive enough to make them competitive, or enable them to immediately impact the workplace with a high degree of efficiency where they can put into practice all of the theory they have learned in the classroom and the expertise they have acquired. Providing avenues for participation in an enrichment graduate education program is essential and critical for maintaining healthy communities [12,13]. It is this type of cognitive intervention, fused with strong mentoring and training, which will set the stage for a successful health intervention and subsequent transformation of the health status among African Americans in Mississippi. 

Recently, there has been support for a paradigm shift towards utilization of learning communities to provide accelerated professional development of public health scholars [14,15]. Learning communities allow institutions to provide expertise in a learning environment where graduate students can be exposed to intensive training in preparation for their service as future professionals [16]. The learning community implemented by GTEC and operating through the DHWS program includes an enrichment curriculum coupled with a mentoring experience so that the scholars can learn theoretical cardiovascular epidemiology concepts, while engaged in practical, hands-on, real world research experiences. GTEC combines didactic lectures with simulated, experiential learning. This process is important in Jackson, Mississippi because, as is mentioned above, Mississippi has one of the largest prevalence of CVD. This means that Mississippi communities have higher morbidity and mortality, lower productivity, and increased health care expenses. Thus, it is an important mission of public health professionals to institute measures to transform this state into a healthy society where each and every individual can enjoy a better quality of life. 

## 2. Objectives

This study reports on the implementation of the Jackson Heart Study GTEC DHWS program that serves as a singular, comprehensive medium to facilitate the development of the identified core competencies. This study describes the methods of the Jackson Heart Study GTEC for delivering this effective, unique, comprehensive training program to achieve stated goals. We provide a description of how the Jackson Heart Study is organizationally structured for realizing our objectives.

## 3. Methods

The Jackson Heart Study has developed and is conducting a two-year graduate training program for African American graduate and health professional students through GTEC, under the auspices of Jackson State University, Jackson, Mississippi. A key component of GTEC is the installation of the DHWS program that utilizes the learning community (LC) concept to provide expert training to the scholars who are selected to participate. The organizational structure of GTEC comprises a staff serving in a variety of capacities. They are described in the Table 1 that follows: 

To complete the learning community structure, GTEC enlists the services of four learning community advisors and four instructors. This team makes up the core Daniel Hale Williams Scholar Program learning community (LC). Additional expertise is acquired periodically as needed from expert investigators from around the United States as the program progresses through its phases. The LC Advisors are selected from the faculty/investigators from the two JHS institutions involved in graduate training, JSU and UMMC.

**Table 1:** Structure of the Jackson Heart Study Graduate Training and Education Center.

Position	Responsibilities
Principal Investigator	Provides overall oversight of GTEC
Program Director	Director of the DHWS Program and all of its activities
Senior Research Scientist	Responsible for managing the scientific productivity of the GTEC
Senior Applications Programmer/Data Manager	Responsible for managing and overseeing data collection, development of protocols, evaluation
Information Technology Specialist	Responsible for graphic design, web-developer, information technology
Administrative Assistant	Responsible for general office management duties
Business Manager/Contract Administrator	Responsibilities include overall management of the GTEC budget

## 4. Results

The day-to-day management of GTEC and the DHWS program is undertaken by its administrators through the organization of a Working Group/Committee structure. Five working groups have been established with distinct functions to ensure that all of the requirements for the GTEC and the DHWSP are effectively developed, implemented, managed, monitored, and evaluated. The GTEC Working Groups are presented in Table 2.

**Table 2:** GTEC Working Groups.

Working Group	Objectives
Forms Development	Develop on-line GTEC Scholar application Create student directory Develop course and program; Evaluations Pre-and post- surveys
Marketing, Recruitment and Retention	Create announcements advertising GTEC Development of recruitment materials Develop student recruitment plan Create website advertisements Newspaper stories about GTEC Establish tracking mechanisms
Curriculum	Develop goals and learning objectives for each GTEC course Create one-hour orientation session for GTEC faculty Interface with instructors to develop course materials
Education and Training	Develop mentor training program for GTEC Learning Community Peers Graduate Student; Instructors/Mentors learning community Advisors
Evaluation	Evaluation of GTEC processes, scholar activities, Instructors, and learning community Advisors

## 5. The Daniel Hale Williams Scholars Program

The overall goals of the GTEC are to: (1) enrich the educational experiences and skills of African American graduate students to prepare them for future careers in academia, industry, and government research settings; (2) enhance the professional skills of graduate students to increase the likelihood of success and completion of graduate school; and (3) increase the interest in, and likelihood of, African American graduate students entering careers in biomedical sciences following degree and program completion. The GTEC is dedicated to achieving these goals by engaging the affiliated faculty, staff, mentors, and institutional leaders. These leaders are a diverse group of multidisciplinary faculty with the depth of experience, dedication, and commitment to the provision of education and research training for graduate students to advance cardiovascular research and improve the quality of health care delivery for underserved and vulnerable populations.

With the administrative staff and Working Groups installed, the first objective of GTEC was to establish a graduate-level training program focused on cardiovascular epidemiology research; we achieved that objective by establishing the DHWS program. The DHWS program utilizes an empirically-tested learning model to develop, implement, and evaluate a two-year graduate program in cardiovascular epidemiology, with an emphasis on social epidemiology and health disparities. 

In developing the DHWS program, we examined the recommendations from the Sullivan Commission as the primary guide to identify, select, implement, evaluate, and maintain the health professional educational training ground and mentorship programs that are critical to improve the racial diversity of the healthcare workforce [17,18]. GTEC believes that exposing graduate students to the DHW Scholars Program has the potential to shape, modify, and ultimately reverse the trend of low representation of racial minorities in health professional careers. The GTEC operates with staff who has expertise in adult learning, and provides mentoring and graduate training in social, behavioral, and clinical sciences, epidemiology, cardiovascular disease; students are engaged in regular interaction with experienced investigators who have a deep familiarity and engagement with the Jackson Heart Study and the state-of-the-art research resources and infrastructure available at Jackson State University and the Jackson Heart Study that would ultimately equip them for success.

This innovative training program recruits full-time students in good standing who are enrolled in Psychology, Sociology, and Public Health graduate programs at Jackson State University and students enrolled in medicine, nursing, pharmacy, and dentistry schools at the University of Mississippi Medical Center. This program is designed to provide a strong foundation in epidemiology, cardiovascular disease, health disparities, and professional development to increase the likelihood of African American graduate students entering careers in biomedical sciences. All course offerings are held in the Jackson Medical Mall, where the School of Public Health of Jackson State University and the Jackson Heart Study are located.

Recruitment of students is done using several media. Announcements are posted on billboards that are strategically located in relation to the targeted campuses (Jackson State University and the University of Mississippi Medical Center). Announcements are posted on the websites of the institutions, as well as on bulletin boards in the departments of the students who are targeted. In addition, announcements are made at all JHS public events, as well as the Universities’ events, the JHS Newsletter, and local news media. The deans, faculty, and staff of the targeted departments also serve as spirited recruiters for the program.

All classes/activities in the DHWS program are considered enrichment activities that are offered to supplement the students’ full-time class load in their graduate programs. Students are recruited and selected with the understanding that they must have two years to devote to the program, they must maintain good academic standing required by their institution, and they must meet all the criteria established by their institution in order to maintain their scholarship.

The first cohort of Daniel Hale Williams Scholars selected for the 2013–2014 academic year comprised three students, the second cohort for the 2014–2015 academic year comprised four students. The third cohort for the 2015–2016 academic year comprised four students. It is expected that by the end of the current contract period in 2018, GTEC will have recruited and trained a total of 17 scholars. Scientists from public health departments and other institutions interact with students and provide training during the orientation and the one week training camp. In addition, the scholars participate in summer training institutes where they engage in research training activities from nationally-known scientists from around the country. The LC advisors are matched with the students based on their familiarity with the JHS data and their research interests. Throughout the two years of scholars’ eligibility, the LC advisors guide the scholars through the manuscript proposal development process, the research process, and the final manuscript development process. 

The evaluation component of the program ensures that GTEC is able to monitor progress, address areas of concern, make necessary adjustments, and measure success of the program. The evaluation comprises a student evaluation of the instructors and the LC advisors, the instructors’ and LC advisors’ evaluation of the students’ progress, as well as an evaluation of all of the DHWSP activities. We have developed a tracking system that will track our scholars to document milestones such as graduation rates and professional employment. Our tracking system will measure long-term success of the scholars and the program. Shorter term outcomes that will be measured include the scholars’ completion of research projects, presentations of research at conferences, completion of proposals using JHS data, submission of the proposals to the JHS P and P for approval, completion of a manuscript, and subsequently publication of a manuscript.

The JHS Graduate Training and Education Center utilizes four key components of core competencies which serve as the basis of the DHWS program: (1) the enrichment curriculum; (2) learning community; (3) quarterly seminar; and (4) Summer Institute. These activities enable GTEC to provide quality cardiovascular epidemiology training to the graduate scholars enrolled in the program. Table 3 represents components of the DHWS program and the activities to which the scholars are exposed.

### 5.1. Enrichment Curriculum

The DHWS program curriculum was developed to train students to design, analyze, and evaluate epidemiologic and related biomedical research; demonstrate an understanding of and ability to, apply biostatistics and other appropriate quantitative research and evaluation methods; and apply current research methods in cardiovascular disease epidemiology. The first year of the program consists of interactive delivery of courses on the design, analysis, and evaluation of epidemiology research, with an emphasis on cardiovascular disease. During the first year, scholars also attend a Summer Research Training Program. The minimal goals for the scholars for Year 1 include: at least 85% attendance at DHWSP activities, timely submission of DHWS program reports, abstract submission, and presentation on Research Day at the Annual Conference on Eliminating Health Disparities Sponsored by the Center for Excellence in Minority Health and Health Disparities at Jackson State University.

During Year 2, the emphasis is on finalizing and submitting a manuscript for publication. The second year is characterized by hands-on opportunities to apply acquired research knowledge using a data set derived from the Jackson Heart Study data. Upon completion of this program, students will receive a certificate of completion. 

### 5.2. Quarterly Seminar Series

The Quarterly Seminar Series focuses on social epidemiology, examining differential exposures and health outcomes among populations. During this series, seminars also include presentations on identifying social interactions, while addressing experimental social epidemiology. Speakers consist of national and local experts, including those from the Jackson State University (JSU) and the University of Mississippi Medical Center (UMMC).

**Table 3:** Program Components and Activities of the DHWS Program.

Program Components/Activities	Program Description
Daniel Hale Williams (DHW) Scholars Orientation	Introduction of DHW scholars and faculty Program overview and expectations Mentor and mentee training
Enrichment Curriculum	Conducting JHS data analysis using appropriate statistical techniques Developing a JHS research proposal Training in biostatistics, epidemiology, and cardiovascular epidemiology
Research Camp	Week-long research camp where the scholars are exposed to lectures by renowned scientists focusing on reading, writing, surviving and thriving in the research arena.
Quarterly Seminar Series	Presentations on social epidemiology, public health, and biomedical sciences
Learning Community Advisor Interaction	One on One mentoring from investigators familiar with the JHS datasets.
Summer Scholar Institute	Summer Research Training Programs are available in public health and biomedical sciences at local and national institutions
JHS Research Day	JHS DHW Scholars from GTEC present findings of their research in either poster or oral format Awards are presented for the top scholar presentations
DHW Brown Bag Seminars	Periodic workshops and seminars highlighting a variety of research topics.

### 5.3. Research Camp

The Research Camp is a one week intensive program, focusing on increasing students’ overall quality and knowledge of research, and improving their oral and written communication skills. It utilizes didactic and interactive approaches. Lectures and exercises address strategies for reading and critiquing the scientific literature; scientific writing; enhancing skills in evaluation and statistics; designing epidemiological studies; and career navigation. 

### 5.4. Learning Community Advisors/Mentoring

Mentoring is a critical element for the advancement and success in academic careers. At GTEC, our scholars are paired with a learning community advisor whose research expertise mirrors their research interest. The learning community advisors guide them through the research process.

### 5.5. Summer Obesity Research Program

Scholars participate in an intensive didactic and interactive summer research program focused on social epidemiology, cardiovascular disease, cardiovascular epidemiology, health disparities/health equity research, and the JHS. Researchers with substantial expertise in these areas deliver the curriculum via in-person, didactic sessions, as well as participate in career mentoring sessions. Faculty from other institutions travel to Jackson to provide training for the scholars during the orientation and the one week long Research Camp, as well as during the Quarterly Seminars. The scholars also travel to participate in Summer Institutes and conferences. Summer training is conducted in collaboration with institutions around the nation, including the University of Michigan, Ann Arbor and Drexel University in Pennsylvania.

## 6. Discussion

The DHWS program began in 2014, and has enrolled three cohorts of scholars to date for a total of 11 students. We anticipate training 17 students by the end of the school year in 2018. The scholars’ success is measured by their completion of the academic training activities, successful interaction with the learning community advisor, completion of 85% of prescribed DHWS program activities, (See Table 4) as well as completion of a manuscript proposal using Jackson Heart Study data leading up to a published manuscript in a peer-reviewed scientific journal. Scholars will continue to be tracked after the two year activity period ends to update their career status, pathways, and opportunities. Jackson State University through the Jackson Heart Study GTEC in the School of Public Health is the only institution in the United States that offers this type of training that complements the regular curriculum in public health. This DHWS program allows students opportunities to develop competency in specific areas of study by providing an opportunity for immersion in the field of cardiovascular epidemiology. Participation in APHA and AHA EPI/Lifestyle meetings are also included in the scholars’ training activities. A number of universities and schools of public health offer certificate programs in epidemiology, but the JHS DHW scholars program is the only US cardiovascular epidemiology certificate program, and is a unique resource, given the importance of CVD as a cause of morbidity and death in the United States.

We began operation of the DHWS program with the expectation that a well-organized learning community is associated with a strong graduate student collective efficacy, hence making our scholars competent for entry in the health care profession. This design is an emerging trend in higher education and professional development, and has been touted as a strategy to increase student success and retention [19,20]. For the Jackson Heart Study, implementing the DHWS program would not only benefit Jackson State University but the community as well. Through the DHWS program, the scholars are actively engaged in academic coursework along with training and interaction with renowned professionals/scholars, and we believe that this approach will eventually produce prepared graduate professionals who are eager and ready to tackle CVD in addition to other health disparities. This program may be used as a model and can be applied to future learning communities interested in training graduate students in cardiovascular epidemiology. Through the DHWS program, our graduate students have become engaged in an enrichment curriculum that is training them to: (1) design, analyze, and evaluate epidemiologic and related biomedical research; (2) demonstrate an understanding of quantitative research and the ability to apply biostatistical and other appropriate quantitative research and evaluation methods; and (3) apply current research methods in cardiovascular disease epidemiology. 

The main strength of the GTEC DHWS program is that the scholars have an opportunity to access data from the largest epidemiological study ever conducted in an African American cohort. The scholars also have the opportunity to interact with Jackson Heart Study investigators on site who are intimately familiar with the data and can assist them with their research development. Through the GTEC DHWS program, they have an opportunity to gain an understanding of emerging risk factors for CVD and the relation of variables that contribute to the prevalence and incidence of those diseases in an African American population. Students also have the opportunity to publish manuscripts and attend and present at major conferences. A limitation of the program is the process for navigating the Jackson Heart Study bureaucratic framework for acquiring data to conduct research. This process is challenging for scholars who have a limited amount of time to complete their assigned tasks, and delays in the approval process for manuscript proposals and acquisition of data could severely limit their ability to meet all of their objectives.

**Table 4:** GTEC DHWS Program/Schedule of Events.

Months and Events			
**August**	**September**	**October**	**November**
**Events**	**Events**	**Events**	**Events**
Seminar 1 JHS Medication Coding & Grant Writing	**800-01** Cardiovascular Disease Epidemiology Seminar 2 Health Disparities Brown Bag Luncheon/Scholars’ Meeting	Health Disparities Conference/Research Day **801-01** Hot Topics in Epidemiology/Health Disparities/Health Equity Brown Bag Luncheon Scholars’ Meeting America Public Health Association Meeting	American Public Health Association Meeting Brown Bag Luncheon/Scholars’ Meeting **802-01** Anatomy and Physiology/Introduction to Social Epidemiology/Research Methods in Social Epidemiology
**December**	**January**	**February**	**March**
**Events**	**Events**	**Events**	**Events**
**803-01** Applied Biostatistics/JHS Data Analysis Brown Bag Luncheon/Scholars’ Meeting	Brown Bag Luncheon/Scholars’ Meeting **800-02** Cardiovascular Disease Epidemiology	**801-02** Hot Topics in Epidemiology/Health Disparities/Health Equity Seminar 3 Epidemiology Brown Bag Luncheon/Scholars’ Meeting	American Heart Association EPI/Lifestyle 2016 Scientific Sessions **802-02** Anatomy and Physiology/Introduction to Social Epidemiology/Research Methods in Social Epidemiology Brown Bag Luncheon/Scholars’ Meeting
**April**	**May**	**June**	**July**
**Events**	**Events**	**Events**	**Events**
Social Epidemiology **803-02** Applied Biostatistics/JHS Date Analysis	Orientation Research Camp	Summer Institute	Summer Institute
Brown Bag Luncheon Scholars’ Meeting Annual Selection of Graduate Training and Education Center Daniel Hale Williams Scholars Cohort	Evaluation of the Program		

## 7. Conclusions

The Jackson Heart Study GTEC DHWS program has a rigorous academic and career-focused curriculum, and its core competencies offer a comprehensive approach to identifying, recruiting, and providing training to graduate students as they further develop the competencies needed to impact cardiovascular disease management programs. 

As mentioned earlier, there is no other institution in the United States that offers a graduate program in cardiovascular epidemiology leading to a certificate of completion. In the State of Mississippi where cardiovascular disease is most rampant, particularly among African Americans, the opportunities provided by the initiation of the DHWS program have the potential to increase the pool of public health and biomedical scientists who can provide services within the Mississippi communities to help alleviate the impact of these diseases and reduce the prevalence of risk factors that eventually lead to the development of CVD. 

The DHWS program goes beyond the foundational and disciplinary-specific material typically presented in these courses in the regular academic curriculum. The enrichment activities are complementary to the core curriculum and are typically designed to further develop research skill sets. GTEC aims to contribute to building evidence-based approaches to training minority graduate students in the social, behavioral, and medical sciences through research by maintaining a network of partners who can implement robust education training programs uniquely tailored to minority students addressing the historic and current challenges experienced by this group. 

The entire program is facilitated by the GTEC investigators’ deep familiarity and engagement with the JHS and the existence of state-of-the-art research resources and infrastructure for leading the GTEC. The GTEC has enjoyed strong successes in the two years of operation and the program has resulted in positive student results. The students enter this formal program with a common purpose and become empowered through their social interaction to complete their courses and other activities as a cohort, while, at the same time, pursuing individual and group learning opportunities. As mentioned by Thompson [21], the scholars learn the virtues of shared decision-making and trust-building and the role these values play in career building, health promotion, and community capacity-building [21].

The GTEC in the School of Public Health at Jackson State University is a very new program. Future plans are to compare milestones, such as graduation rates of the GTEC scholars and the entry of the scholars into professional employment. We have already compiled a list of presentations completed by the scholars from the first three cohorts at conferences, as well as publications that they have completed. Plans are in place to compare the scholars’ scientific productivity, as well.

## References

[1] American Heart Association (2004). Heart Disease and Stroke Statistics-2004 Update.

[2] Campbell-Jenkins B.W., Addison C.C., Young L., Anugu A., Wilson G., Sarpong D. (2009). Development of the Jackson Heart Study Coordinating Center. Int. J. Environ. Res. Public Health.

[3] The National Heart, Lung, and Blood Institute (2008). The Jackson Heart Study Data Book: A Report to the Cohort and Community.

[4] Robert Wood Johnson Foundation State of Obesity in America. http://stateofobesity.org/.

[5] Dubbert P.M., Robinson J.C., Sung J.H., Ainsworth B.E., Wyatt S.B., Carithers T., Newton R., Rhudy J.L., Barbour K., Sternfeld B. (2010). Physical activity and obesity in African Americans: The Jackson Heart Study. Ethn. Dis..

[6] Finkelstein E.A., Trogdon J.G., Cohen J.W., Dietz W. (2009). Estimates annual medical spending attributable to obesity: Payer and service. Specif. Health Aff..

[7] Heffler S., Smith S., Won G., Clemens M.K., Keehan S., Zezza M. (2002). Health spending projections for 2001–2011: The latest outlook. Health Affair..

[8] The National Academies Press (2003). Front Matter Unequal Treatment: Confronting Racial and Ethnic Disparities in Health Care.

[9] Srinivasan A., Brown J., Fahmy N., Heitman E., Singh M., Szklo M., Taylor H., White W. (2005). Preparing African Americans for careers in health care: The Jackson Heart Study. Ethn. Dis..

[10] Tremethick M.J., Smith E.M. (2009). Preparing culturally competent health educators: The development and evaluation of a cultural-immersion-training program. Int. Electron. J. Health Educ..

[11] Jones L., Stall G., Yarbrough D. (2013). The importance of Professional Learning Communities for school improvement. Creative Educ..

[12] Lee J.C., Zhang Z., Yin H. (2011). A multilevel analysis of the impact of a Professional Learning Community, faculty trust in colleagues and collective efficacy on how incorporating a Learning Community will prepare. Teach. Teach. Educ..

[13] Manzuk D., Clifton R.A. (2008). Stages of collaboration and the realities of professional learning communities. Teach. Teach. Educ..

[14] Carr P.L., Bickel J., Inui T.S. (2004). Taking Root in a Forest Clearing: A Resource Guide for Medical Faculty.

[15] Vescio V., Ross D., Adams A. (2008). A review of research on the impact of Learning Professional Community on teaching practice and student learning. Teach. Teach. Educ..

[16] Darling-Hammond L., McLaughlin M.W. (1995). Policies that support professional development in an era of reform. Phi Delta Kappan.

[17] The Sullivan Commission (2004). Missing Persons: Minorities in the Health Professions.

[18] Smedley B.D., Stith A.Y., Colburn L., Bristow L.R. (2004). The Nation’s Compelling Interest: Ensuring Diversity in the Healthcare Workforce.

[19] Li L.C., Grimshaw J.M., Nielsen C., Judd M., Coyte P.C., Graham I.D. (2009). Evolution of Wenger’s concept of community of practice. Implement. Sci..

[20] Tinto V. (2003). Learning Better Together: The Impact of Learning Communities on Student Success.

[21] Thompson S.P. A Multiple Case Study of Professional and Personal Outcomes of a Professional Development Doctoral Program with Learning Community Base. Doctoral Dissertation.

